# Health related quality of life, service utilization and costs for patients with Huntington’s disease in Norway

**DOI:** 10.1186/s12913-022-08881-8

**Published:** 2022-12-14

**Authors:** Marleen R. van Walsem, Jan C. Frich, Monica Gómez Castañeda, Emilie Isager Howe, Lasse Pihlstrøm, Nada Andelic, Eline Aas

**Affiliations:** 1 Department of Neurohabilitation, Oslo University Hospital, P.O. Box 4950 Nydalen, 0424 Oslo, Norway; 2 Department of Neurology, Oslo University Hospital, P.O. Box 4950 Nydalen, 0424 Oslo, Norway; 3 Institute of Health and Society, CHARM - Research Centre of Habilitation and Rehabilitation Models and Services, Univeristy of Oslo, P.O. Box 1130 Blindern, 0318 Oslo, Norway; 4 Institute of Health and Society, University of Oslo, P.O. Box 1130 Blindern, 0318 Oslo, Norway; 5 Division for Health Services, Norwegian Institute of Public Health, Oslo, Norway; 6 Department of Physical Medicine and Rehabilitation, Oslo University Hospital, P.O. Box 4950 Nydalen, 0424 Oslo, Norway

**Keywords:** Huntington’s disease, Utilization, Health care and societal costs, Health-related quality of life

## Abstract

**Background:**

Huntington’s disease (HD) is a progressive genetic neurodegenerative disease accompanied by mental and neurocognitive disabilities, which requires long-term and comprehensive treatment and care. Information on the health and economic burden of HD is scarce, but essential for conducting health economic analyses, in light of the prospect of new therapies for HD. In this study, we aim to identify values for Health-Related Quality of Life (HRQoL), describe service utilization and costs, and their associations with clinical and socio-demographic variables across all phases of HD.

**Methods:**

A cross-sectional study including 86 patients across all phases of HD. Values of HRQoL were calculated based on EQ-5D-3L index scores. Additionally, health care and societal costs were estimated based on service utilization collected using the Client Service Receipt Inventory (CSRI) and data from the patients’ interviews. Total societal costs included costs of primary and secondary health care services, informal care and productivity loss of the patients. Multiple regression analyses were used to investigate associations between socio-demographic and clinical variables on HRQoL and costs.

**Results:**

HRQoL values declined, while total costs increased across disease severity. Total six-month healthcare costs and total societal costs were € 18,538 and € 66,789 respectively. Healthcare and societal costs doubled from early to middle phase, and tripled from middle to advanced disease phase. Main six-month cost components for the three disease phases were informal care costs (€ 30,605) accounting for approximately half the total societal costs, and costs due to production loss (€ 18,907) being slightly higher than the total healthcare costs. Disease severity and gender were found to have the strongest effect on both values of HRQoL and costs.

**Conclusions:**

Reported values of HRQoL and costs including costs for production loss may be used in modelling the cost-effectiveness of treatment for HD. Our results highlight the crucial role the informal caregivers play in the care provided to HD patients in all disease phases. Future research should focus on the estimation of productivity loss among informal caregivers.

**Supplementary Information:**

The online version contains supplementary material available at 10.1186/s12913-022-08881-8.

## Introduction

Huntington’s disease (HD) is a genetic neurodegenerative disorder, caused by an expanded CAG repeat in the *HTT* gene located on chromosome 4, affecting people in the middle of adult life usually between 30 and 50 years of age [1, 2]. HD is a rare disorder with prevalence estimated at 3.6–5.7 in populations of European ancestry [3, 4], characterized by movement disorders, specifically chorea (dance-like) movements, a variety of mental symptoms such as mood disturbances, irritability and apathy, as well as a decline in cognitive function resulting in dementia [2, 5].

The course of HD requires complex long term multidisciplinary treatment and care in the absence of disease modifying treatments [2, 6–8]. HD has substantial impact on patients’ and carers’ lives as well as the health and social care systems [9–12]. Despite this, information about the health and economic burden of HD is scarce. The prospect of new therapies for HD, and other genetic diseases with no previous effective disease modifying treatment, makes health economics analyses highly warranted.

Studies on health related quality of life (HRQoL) in patients with HD based on generic measurements (i.e. Short Form Health Survey-36, EuroQol [13, 14]) and condition specific measurements (i.e. Huntington’s Disease health-related Quality of Life questionnaire) generally indicate lower HRQoL in patients with clinical HD compared to individuals with premotor manifest HD and individuals at risk. HRQoL scores decline with increased disease severity and correlate most strongly with neuropsychiatric and cognitive symptoms [15–18]. One recent European study found that HRQoL values (SF-6D) were generally lower for women compared to men [19]. Furthermore, the study found that HRQoL values declined with time since diagnosis, behavioural symptoms, increasing age, and disease severity [19]. Busse et al. showed that despite that most patients in Europe utilize formal care services, there was a large reliance on informal care (care provided by non-professionals such as family or friends) [11]. A limited number of studies conducted in specific countries across the world, has investigated the economic burden of HD by calculating costs of HD based on participant reports of healthcare service utilization, informal care provision, and data from insurance claims [20–23]. They found that costs increased across disease stages, with highest costs for patients with advanced HD [20–22]. Moreover, informal care provision was found to be the largest driver of costs, and health care services costs were highest for outpatient services [20, 22]. To our knowledge, there is no study investigating the economic burden of HD in Norway or in any Nordic country.

In the present paper we aim to a) describe HRQoL values using EQ-5D-3L across all phases of HD measured by Unified Huntington’s Disease Rating Scale – total functional capacity scale, b) describe service utilization, c) to assess the costs and cost composition for HD, based on information of service utilization, and d) to explore associations between EQ-5D-3L estimates, costs, socio-demographic and clinical characteristics in a cross-sectional study of a Norwegian population of patients with HD across early to advanced HD.

## Method

### Participants and recruitment

In the present cross-sectional study, eligible patients that a) had a clinical diagnosis of HD and b) resided in the South-Eastern region of Norway were identified and invited through Oslo University Hospital and rehabilitation centres with programs for HD patients. All eligible patients received a written invitation, containing study information and an informed consent form. After receiving the signed consent form, the patient or carer was contacted and an appointment for the study visit was scheduled. Out of 158 eligible patients, 88 patients consented to participate in a survey of healthcare service utilisation and needs for healthcare services. Two out of the 88 participants were excluded because clinical diagnostic criteria were not fully met, resulting in 86 out of 158 (54.4%) participants being included in the analyses. Two patients did not return a HRQoL questionnaire (EQ-5D-3L), resulting in 84 patients included in analyses of HRQoL data.

### Ethics

The study was approved by the Regional Committees for Medical and Health Research Ethics South East Norway (ref. 2013/2089) and performed in accordance with the Declaration of Helsinki. For participants who were unable to provide informed consent themselves, consent was obtained from the primary caregiver or legal representative.

### Data collection, procedures and measures

Two clinical raters, experienced in the field of HD (MRvW and EIH), collected data from January to August 2014 through survey interviews during an outpatient study visit (38%) or at the patients’ home (62%). About one third of the visits were conducted with the patient alone (31%), while the remaining interviews were done with the patient and primary informal and/or formal carer, or with the informant only (69%). Background information including socio-demographic and clinical information and disease characteristics were recorded. We calculated disease duration in years as the date of formally obtained clinical diagnosis of HD subtracted by the date of the study visit. Moreover, a clinical functional evaluation was performed, assessment of needs was conducted, and the use of healthcare services was recorded. At the end of the study visit we requested patients to complete the EQ-5D-3L. Primary carers assisted participants who were unable to fill out the questionnaire independently and were specifically informed to assist reflecting the participants’ own rating of their health status, as this is a self-report measure.

#### Unified Huntington’s disease rating scale (UHDRS) – functional assessment

In order to assess participants’ functional status and disease phase, we used the UHDRS-Functional assessment [24] comprising three scales. First, the Total Functional Capacity Scale (UHDRS-TFC), rating the ability to engage in occupation, manage finances and domestic chores, and to perform activities of daily living (ADL). It has a score range of 0–13, and the scale is used to classify patients into five functional disease stages or three disease phases. The early phase comprises stages I and II represented by TFC scores of 11–13 and 7–10, respectively, the middle phase is represented by stage III and a TFC score of 3–6, and the advanced phase includes disease stages IV and V with TFC scores of 1–2 and 0. Second, the Functional Assessment Scale (FAS), a daily living checklist with scores ranging from 0 to 25. Third, the Independence scale (IS) with score range from 10 to 100 indicating overall functional independence. Higher scores on these scales indicate better functioning [25].

#### HRQoL

In order to assess study participants’ HRQoL and to calculate individual HRQoL values, the three-level EuroQol five-dimensional questionnaire (EQ-5D-3L) was used [13]. The EQ-5D-3L scale assesses HRQoL across five dimensions: *Mobility*, *Self-Care, Usual Activities, Pain/Discomfort and Anxiety/Depression*, and within each dimension there are three levels of severity: *no problems, some problems* and *extreme problems,* summarizing to 243 possible health states. Patients are asked to report the level for each of the 5 dimensions, which describes the patients’ current health state. Each health state is assigned a HRQoL value between 0 and 1, reflecting the severity of the health state, where 0 refers to death and 1 to perfect health. The Danish tariff was used to estimate the HRQoL values, as a Norwegian tariff is currently not available (EuroQol, 2020).

#### Service utilization and costs

Data on healthcare and social services utilization were recorded using the Client Services Receipt Inventory (CSRI) [26]. The CSRI measures service utilization the last 6 months, and is a widely used scale in studies on mental health outreach services, community services and community care and has also been used in HD research, including in the EHDN REGISTRY study and Enroll-HD [11, 26]. The questionnaire is filled out by the researcher together with the person receiving services assisted by their main carer when required. For each received service type, the number and average duration of contact is recorded for a fixed (depending on the type of research) retrospective period of time and enables to summarize specific care packages, show the variety of services used and determine how services should be allocated. The CRSI covers a broad range of services that may be utilized including primary and secondary care services, other services, aids as well as informal care provided (care provided by non-professionals such as family or friends). In addition, the version used in this study allows to record service utilization specifically due to HD and related to other health issues during the past 6 months [27]. Furthermore, the CSRI is suitable for calculating cost estimates as it records health care service utilization in detail [26].

Service utilization costs were calculated based on the resource use estimates for a 6 month period as measured by CSRI. Costs and resource utilization related to HD were included. Health care costs were categorized as *Primary care* (general practitioner and physiotherapist), *Home care* (practical assistant and nurse at home), *Nursing homes, Specialists* (specialists outside hospital, such as psychologists, psychiatrists, imaging, MRI, EEG, CT, family therapists and nutrition) or *Secondary care* (hospital services divided into outpatient specialist visits and hospital stays). We estimated the cost of *Informal care*, *Social worker* and *Out-of-pocket* (which included acupuncture, aromatherapy, foot zone therapy, dentist, chiropractor and foot care). Lastly, we included *production loss* for the patient.

The unit costs of primary healthcare services, imaging costs and laboratory tests (i.e., MRI, EEG, CT and blood test) were obtained from the List of reimbursement fees (Normaltariffen) for 2019. The unit costs for outpatient visits and hospital admissions were based on the diagnosis related groups (DRGs). Each visit or admission was assigned a DRG weight reflecting the resource need relative to an average hospital patient. The unit cost was derived by multiplying the DRG weight with per DRG point. Informal care categories were taken from the CSRI. These costs were calculated as the average hourly wage rate multiplied by the number of hours of care per week and scaled up to 6 months (Statistics Norway 2019). Cost of nursing homes for a six-month period were based on average costs in a Norwegian database (KOSTRA) with administrative information on municipal and county activities (Statistics Norway, 2019). To estimate the out-of-pocket costs for services, such as dental care, aromatherapy, foot zone therapy and foot care, we used prices reported online. The unit costs in Euro’s based on a conversion rate of 10.6 NOK per Euro, are reported in Table 1.

**Table 1:** Overview of unit costs and source per service

Service	Unit cost (€)	Source
**Primary care**		
General Practitioner (visit)	42.5	Helfo, 2020
Physiotherapist (30 min)	58.3	Helfo, 2020
Speech therapist (hour)	92.5	Estimate
**Home care**		
Home nursing (hour)	47.2	Real costs
Practical assistance (hour)	37.7	Estimate
**Nursing home** (6 months)	45,827	KOSTRA, 2018
**Rehabilitation** (per day)	245	Real Charge
**Specialists**		
Counselor (genetic)	23.4	Normaltariffen,2019
Family therapy	212	Normaltariffen,2019
Nutritionist	212	DRG 910O
Psychiatrist	115	Normaltariffen,2019
Psychologist	23.4	Normaltariffen,2019
Nurse	35.8	Normaltariffen,2019
Genetic test	23.4	Normaltariffen,2019
MRI	274	Normaltariffen,2019
CT	217	Normaltariffen,2019
Blood test	8.3	Normaltariffen,2019
**Secondary care**		
Neurology OP	315	DRG 901O
Other OP	264	DRG 877O
Day visit Neurologist	834	DRG 980A
**Hospital admissions**		
Hospital admission Neuro.	3220	DRG 35
Hospital admission IC	5735	DRG 12
Hospital admission Other dpt.	5199	DRG 34
**Informal care** (hour)	39.0	Reported wage rate
**Other – out of pocket**		
Acupuncture (1/follow-up visits)	75.5/51.9	Estimate^a^
Aroma therapy	66.0	Real costs
Foot zone therapy	55.7	Real costs
**Social worker**	37.7	Estimate

Note. ^a^ = only one patient received 6 consultations of acupuncture, these were priced as the standard acupuncture treatment which consists of 6 visits. Out of pocket payment. *OP* Outpatient, *IC* Intensive care, *DRG* Diagnose related group

Costs related to production loss were calculated as the difference between the six-month salary of a patient with HD and that of the general population in the same age group (Statistics Norway, 2020), adjusted for the proportion in part-time positions. Production loss was not estimated for patients older than 65 years, as this is the retirement age in Norway. To estimate the monthly salary of the study population, working hours were calculated and adjusted to each patients’ percent of a full- time position (i.e., 160 hours per month). The working hours were then multiplied by the age-adjusted hourly rate to estimate the monthly earnings and scaled up to 6 months. An overview of the workforce and earnings for patients with HD and for the general population are shown in Additional file 1.

### Statistical analysis

#### Descriptive analyses

The socio-demographic and clinical disease characteristics served as independent variables. They were reported by means of descriptive statistics (proportions, mean values and standard deviations) across the complete sample and across the three disease phases. Marital status group “single” included participants who were single, widowed, or separated, while the married group included those who were married of partner. HRQoL values across disease stages were also described using mean values and standard deviations. Healthcare service utilization as recorded by the CSRI was presented using the proportion of participants that used the different healthcare services divided according to use due to HD and due to other conditions. Type of informal care provided to patients was reported using proportions and hours per week. All calculated costs are presented using descriptive statistics of mean values with standard deviation and range for the above described cost groups for the whole population and across the three disease phases (disease severity).

#### Regression analyses

Univariate and multiple regression analyses were applied to investigate associations between disease severity, represented by disease phase and disease duration and HRQoL values and costs. As costs are non-negative and typically right skewed, we applied a log-linear regression for costs. To adjust for underlying health condition and risk, patient characteristics such as age, gender, comorbidities and marital status were included. Goodness of fit statistics (adjusted R-squared) were calculated for the multiple regression models. All analyses were conducted using STATA 16.1.

## Results

### Description of the participants

Patient and clinical characteristics across disease phases (early, middle and advanced) are reported in Table 2. Participants’ average age was 57 (SD = 11.4), with age increasing across disease phases. Most of the participants were male, married, lived at home, and had < 12 years of education. The division of occupation (manual or non-manual) was equal for the complete sample. For the total sample, average disease duration was 7.2 years (SD 4.2). Clinical disease characteristics of the patients in the sample distributed as expected, with longer disease duration and decreasing functional scores across disease stages. Regarding other comorbidities, most of the participants (57%) had no comorbidities.

**Table 2:** Population characteristics for the total sample and according to HD stage. Numbers are frequencies and percentages, if not stated otherwise

Variables	Categories	Complete sample	Early HD (TFC 7–13)	Moderate HD (TFC 3–6)	Advanced HD (TFC 0–2)
		(***N*** = 86)	(***n*** = 35)	(***n*** = 19)	(***n*** = 32)
**Socio-demographic variables**					
Age, mean (SD) range		57 (11.4) 28–87	53 (11.9) 28–71	59 (11.1) 39–82	59 (10.2) 41–87
Gender	Female	39 (45)	13 (38)	7 (37)	17 (55)
	Male	47 (55)	21(62)	12 (63)	14 (45)
Occupation	Manual	40 (49)	14 (41)	12 (63)	14 (50)
	Non Manual	41 (51)	20 (58)	7 (37)	14 (50)
Education	< 12 years	52 (60)	16 (47)	15 (79)	20 (65)
	12+	34 (40)	18 (53)	4 (21)	11 (36)
Housing situation	Living at home	54 (63)	34 (100)	13 (68)	5 (16)
	Not living at home	32 (37)	–	6 (32)	26 (84)
Resindence	Rural	13 (15)	4 (12)	2 (11)	6 (19)
	Urban	73 (85)	30 (88)	17 (90)	25 (81)
Marital status	Married (married and living together)	50 (58)	23 (68)	10 (53)	15 (48)
	Single (single, separated, widowed)	36 (42)	11 (32)	9 (47)	16 (52)
**Clinical and disease characteristics**					
Disease duration, mean (SD) range		7.2 (4.7) 0.7–18.7	4.6 (4.1) 0.7–18.7	7.0 (3.8) 1.1–17	10.2 (4.1) 4.3–17.6
Total FAS score, mean (SD) range		12.7 (8.7 0–25	21.4 (2.6) 15–25	13.6 (2.9) 7–18	2.7 (2.8) 0–9
Independence score, mean (SD) range		60.1 (26.3) 10–100	85.0 (9.0) 75–100	64.7 (6.3) 50–70	29.7 (22.8) 10–50
Comorbidity	None	50 (57)	16 (47)	9 (47)	23 (74)
	1 or more	36 (43)	18 (53)	10 (53)	8 (26)

### HRQoL values and associations

Mean HRQoL values declined across disease phases for the 84 participants; early phase (*n* = 34): mean = 0.84 (SD = 0.13), middle phase (*n* = 19): mean = 0.74 (SD = 0.10) and advanced phase (*n* = 31): mean = 0.65 (SD = 0.5). Results of the univariate and multiple regressions are displayed in Table 3. In both the univariate and multiple regression analyses, we found statistically significant negative associations between EQ-5D-3L estimates and disease duration (*p* = 0.050) and disease progression from early to middle HD (*p* = 0.003) and from early to advanced HD (*p* < 0.001), indicating that both longer duration of disease and disease progression was associated with lower HRQoL values. We found a significant association* between higher HRQoL values and male gender (*p* = 0.045). Univariate regression analyses further showed a significant association between HRQoL values and marital status indicating slightly higher values for married participants (*p* = 0.029*)*. However, this association disappeared in the multiple regression analyses.

**Table 3:** Univariate and Multiple Regression Analyses Results for HRQoL values, using EQ-5D-3L

Variable	Category	EQ-5D-3L estimates	
		Univariate	Multiple
		Coef. (SE)	Coef. (SE)
Age group	< 50	Reference	
	50–60	−0.05 (0.04)	0.01 (0.03)
	> 60	−0.02 (0.04)	0.04 (0.03)
Gender	Female	Reference	
	Male	0.07* (0.03)	0.05* (0.02)
Disease phase	Early	Reference	
	Middle	−0.11*** (0.03)	−0.09*** (0.03)
	Advanced	−0.19*** (0.37)	−0.15*** (0.03)
Disease duration		−0.01*** (0.003)	−0.006* (0.003)
Comorbidities	No	Reference	
	Yes	0.004 (0.03)	−0.04 (0.02)
Marital status	Single	Reference	
	Married	0.06* (0.03)	0.03 (0.03)
Constant			0.83*** (0.03)
Adjusted R^2^			0.45

* = *p* ≤ 0.05. ** = *p* ≤ 0.01. *** = *p* ≤ 0.001

### Service utilization

Table 4 provides an overview of the service utilization related to HD. Overall, during the last 6 months, the most frequent used services (average proportion per individual in the sample) were GP visits (0.42), practical assistance (0.51) and conducting blood tests (0.58). Furthermore, among patients using a service, service utilization for physiotherapy with 24.86 visits, speech therapy with 19.47 visits, home nursing 183 days and practical assistance 21 hours per week summing up to 546 hours in 6 months, were most frequently used.

**Table 4:** Service Utilization related to HD and Other Disease in the Study Population using the CSRI (recorded over 6 months, if other not reported)

Type of service (visits)	HD patient service utilization	
	Mean (SD) for patients to use the service	Average proportion per individual in the sample using the service
**Primary care (community care)**		
GP	3.42 (4.17)	0.42
Physiotherapist	24.86 (18.46)	0.33
Speech therapist	19.47 (13.51)	0.20
**Home care**		
Home nurse (days)	182.73 (403.60)	0.17
Practical assistance (hours per week)	21.28 (85.99)	0.51
**Nursing home (months)**	6.00 (−)	0.21
**Rehabilitation (days per 6 months)**	31.5 (−)	0.37
**Specialists**		
Counselor (genetic)	7.33 (10.97)	0.03
Family therapy	3.00 (−)	0.01
Nutrition	2.00 (−)	0.01
Nurse	6.87 (8.77)	0.17
Psychiatrist	12.00 (21.34)	0.06
Psychologist	7.33 (4.16)	0.03
Genetic test	1.00 (−)	0.01
MRI	1.00 (−)	0.03
CT	1.00 (−)	0.05
Blood	1.08 (0.44)	0.58
**Secondary care**		
Neurology outpatient visit	1.41 (1.53)	0.26
Other outpatient visits	1.70 (1.69)	0.23
**Hospital admissions**		
Hospital Intensive care	1.00 (−)	0.02
Hospital Neuro department	5.00 (−)	0.01
Hospital other department	5.33 (5.13)	0.07
**Out-of-pocket**		
Acupuncture	6.00 (*−*)	0.01
Aroma therapy	3.00 (*−*)	0.01
Foot zone therapy	1.00 (*−*)	0.01
**Social worker**	3.00 (*−*)	0.01

Further, we found that between 22 and 42% of our participants received some form of informal care in addition to formal resource utilization (Personal care = 29%, Help at home = 42%, Help outside the home = 22% and Other help = 23%). The average hours per week used by informal carers to provide this care varied from 1.5 hours for help outside the home to up to the equivalent of two working days based on a 7.5 our day (15 hours per week) for personal care with great variation (Personal care: mean = 15 hours per week (SD = 63), Help at home: mean = 11 hours per week (SD = 62.5), Help outside the home: mean = 1.5 hours per week (SD = 3.6), Other help: mean = 2.3 hours per week (SD = 8)).

### Description of costs

Total healthcare costs, care costs and societal costs related to HD are reported according to cost category in Table 5. From a societal perspective, informal care was the main component of costs, followed by production loss and nursing home care, which also all increased across disease phases (table 5). The six-month healthcare costs and total societal costs was € 18,538 and € 66,789 respectively.

**Table 5:** Average Total health care costs divided for types of costs, care costs and societal costs for HD in Euros (10.6 exchange rate) for the pasts 6 months based in the complete sample (*n* = 86)

Type of care	Six-month HD costs	
	Mean	SD
Primary care (*n* = 85)	1227	2138
Home care (*n =* 86)	1796	8416
Nursing homes (*n* = 86)	9592	18,752
Rehabilitation (*n* = 84)	2848	3746
Secondary care (*n* = 85)	239	429
Specialists (*n* = 86)	190	696
Hospital (*n* = 86)	2255	10,016
**Total Health Care costs (*n*** **= 84)**^**a**^	18,538	22,423
Out-of-pocket (*n* = 85)	83	244
Informal care (*n* = 84)	30,605	127,820
**Total Care costs (*n*** **= 82)**^**b**^	47,594	136,682
Social services (*n* = 85)	1	12
Production loss (*n* = 84)	18,907	13,734
**Total Societal costs (*n*** **= 80)**^**c**^	66,789	139,334

^a^Include primary care, home care, nursing home care, rehabilitation, secondary care, specialists and hospital care ^b^includes total health care costs, out-of-pocket and informal care, ^c^includes all social services and production loss in addition to all other cost types reflected in total health care costs and total care costs

Further looking at costs across the three disease phases, total healthcare costs more than doubled from early to middle phase and more than doubled again from middle to advanced disease phase. Total care costs showed a similar pattern but tripled from early to middle phase and were eight times higher in advanced phase compared to middle phase. As for total societal costs, costs doubled from early to middle phase, and more than tripled from middle to advanced phase disease (see additional file 2). Main six-month cost components for the three disease phases were informal care costs (€ 30,605) accounting for approximately half the total societal costs, and costs due to production loss (€ 18,907) being slightly higher than the total healthcare costs.

**Fig. 1:**
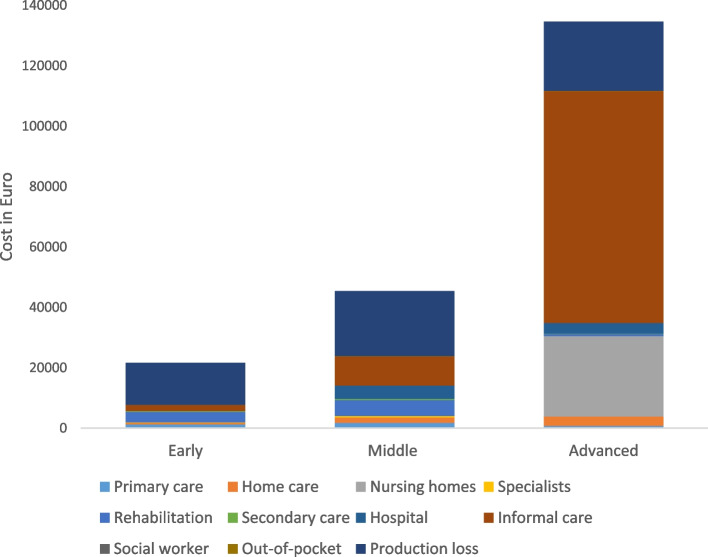
Six-month costs according to HD severity and cost category

Fig. 1 shows the composition of costs for the three disease phases (early, middle and advanced), and illustrates the magnitude of cost composition (see additional file 2 for supporting table). The main cost components for HD in patients in the early phase are (highest to lowest) production loss, rehabilitation, and informal care, for the middle phase production loss, informal care and rehabilitation and, for the advanced phase informal care, nursing homes and production loss (Fig. 1). For several cost components we observe zero costs; nursing homes in early and middle phase, hospitalizations in early phase, and social work in middle and advanced phase. (Fig. 1).

The composition of costs according to marital status and disease phase is reported in Additional file 3. The main observations were that costs were quite similar over cost categories and that married patients in every HD phase had higher costs related to informal care compared to single patients.

### Associations between patient and disease characteristics and costs

Table 6 shows the results from univariate and multiple regression analyses on HD related healthcare costs and societal costs. For healthcare costs we see that in the univariate analyses, costs increased significantly with age (50 to 60 and 60+ relative to < 50) and disease duration, while being in the middle phase implied significant higher costs relative to the early phase of HD, and costs in the advanced phase were significantly higher than both middle and early phases of HD. In the multiple regression analysis, the effects of age and being in the advanced phase on total health care costs remained significant. The univariate analysis on total societal costs showed that being in the age group 50 to 60 years implied significantly higher costs, while males had significantly lower costs compared to females. Similar to health care costs, being in the moderate and advanced phase implied higher societal costs compared to early and moderate phase, respectively. Furthermore, disease duration showed a positive association with total societal costs, while having other comorbidities implied significantly lower costs. In the multiple regression analysis, only the effect on disease phase and gender remained significant for total societal costs.

**Table 6:** Univariate and multiple regression analyses for HD related health care costs

		Total Health Care costs^**a**^			(SE)	Total Societal costs^**b**^		Coef.	SE
		Coef.	(SE)	Coef.		Coef.	(SE)		
**Variable**	**Category**	**Uni**		**Multi**		**Uni**		**Multi**	
**Age group**	< 50 (ref)								
	50–60	1.57**	(0.52)	1.07*	(0.49)	1.22*	(0.48)	0.33	(0.42)
	> 60	1.28*	(0.50)	1.03*	(0.47)	0.24	(0.47)	−0.24	(0.40)
**Gender**	Female (ref)								
	Male	−0.50	(0.43)	− 0.15	(0.35)	− 0.96**	(0.36)	− 0.75*	(0.30)
**Stage**	Early (ref)								
	Moderate	0.89*	(0.44)	0.77	(0.46)	0.92**	(0.39)	0.80*	(0.39)
	Advanced	2.62***	(0.39)	2.62***	(0.47)	2.31***	(0.34)	1.85***	(0.41)
**Disease duration**		0.11*	(0.04)	−0.04	(0.04)	0.13***	(0.04)	0.04	(0.04)
**Comorbidities**	No (ref)								
	Yes	−0.37	(0.43)	0.11	(0.39)	−0.78**	(0.37)	−0.18	(0.33)
**Marital status**	Single								
	Married	−0.34	(0.42)	0.03	(0.36)	−0.59	(0.37)	−0.001	(0.30)
**Constant**				9.03**	(0.53)			11.84***	(0.45)
**Adj R-sq.**				0.36				0.40	

Uni = univariate. Multi = multiple. * = *p* ≤ 0.05. ** = *p* ≤ 0.01. *** = *p* ≤ 0.001. ^a^Include primary care, home care, nursing home care, rehabilitation, secondary care, specialists and hospital care, ^b^includes all social services and production loss in addition to all other cost types reflected in total health care costs and total care costs

## Discussion

This study provides a descriptive analysis of HQRoL values based on EQ-5D-3L and costs incurred by patients with HD from recorded services utilization and data related to workforce for patients with HD, across all three disease phases. We further investigated relationships between patient characteristics with HRQoL values and costs estimates.

We found that EQ-5D-3L values declined by increasing disability and disease severity as represented by disease phase and were found below HRQoL values for normal Norwegian population in middle and advanced phases [28]. These findings were supported in regression analyses where the relationship between disease severity and HRQoL was found to be the strongest among all variables. Additionally, disease duration and gender remained significant in the multiple regression analyses. The gender-driven difference found in this study might reflect that more male patients live at home rather than at institutions as they still have a living spouse due to a longer life-expectancy among females. For females, we therefore find that they are more likely to be institutionalized due to less access of informal care. Further, a gender difference in advance of males is in line with HRQoL values for the Norwegian population [28]. Our findings regarding disease severity and gender differences are also comparable to the findings of the longitudinal European study of Hawton et al., showing lower HRQoL values for patients in advanced and middle phases compared to early phases, with lower values for women [19]. Contrary to results from Hawton et al. and the study of the general Norwegian population [19, 28] we did not find a significant association between age and HRQoL, a result that was confirmed in analyses including age as a continuous variable. This effect may be masked by variables of disease phase and disease duration. Our findings regarding overall service utilization for primary care services of physiotherapy and speech therapy are comparable to results of Busse et al. and of Ohlmeier et al. [11, 23]. Further, the results regarding service utilization supports earlier findings that there is a large reliance on informal care services [11]. This finding underlines the impact of HD on family caregivers and the necessity of providing support to the family [29, 30].

In line with our expectations, the six-month costs increased across HD phase (severity) with the total societal costs being about twice as high when comparing the early (€ 22,005) to the middle phase (€ 40,817), and approximately 3.5 times higher from the middle phase to the advanced phase (€ 133,222). This increase of costs for all three cost groups (total health care, total care and total societal costs) across disease phases is in line with other available studies on estimated costs and economic burden of HD and reflect increasing service utilization of HD patients across disease phases reported in other studies [11, 20–22]. Furthermore, total societal costs were mainly driven by informal care costs followed by production loss costs, care in nursing homes and rehabilitation costs. The large contribution of informal care costs is in line with findings in other studies that included informal care in cost estimations [11, 20, 22]. Similar to the study by Jones et al. 2016, our results show that already in the early phase of HD, informal care costs are higher than other types of health care costs, and these costs steadily increase across phases and become very high in advanced HD [20]. However, we found total informal care costs to be higher compared to Jones et al., with total informal care costs for the full sample of € 30,605 for 6 months compared to £14,085 annually. Jones et al. found slightly higher informal care costs for the middle phase of HD of £21,051 compared to €19,200 for annual costs in our study. Higher overall costs in our study may be explained by the sample consisting of a relatively large group of patients in advanced phase (22%) compared to slightly under 10% in the UK study. We also found informal care costs to be higher for married patients in every phase of HD compared to single patients. Informal care seems a substitute of institutional care (care in nursing homes) as costs related to informal care were higher than nursing home costs in the advanced phase of disease in our study. These results highlight the important contribution of the informal care provided by partners and other family members or friends to the treatment and care of HD patients, especially in late stages of disease, also proposed by previous studies [11, 20]. Despite the high reliance on informal care being as expected and in keeping of findings of previous studies, they may be considered as high in light of Norway being a well-fare state where one may expect that the majority of healthcare provision is offered by a formal care provider.

In addition to informal care, production loss constituted a large proportion of costs with €18,907 on average for the complete sample, and especially high in the early and middle phases of disease. Production loss being the largest cost component, about double of the total cumulative cost types in the early phase of HD is striking. This may point to the effect of early HD symptoms on work capacity and may also indicate that health care and support needs are unmet and not provided. Overall, this confirms the severity of HD and its remarkable impact on the workforce and disability rates. To our knowledge, no previous studies have included estimations of production loss based on data regarding patients’ work-force.

The present study also shows that the majority of various types of healthcare costs reported are highest in the middle phase of HD compared to both early and advanced phase HD. This may reflect a higher need for broad healthcare services reflected by the wide variety in symptom presentation and progression in this phase. These patients are likely to benefit from more medical interventions, compared to early and advanced phases [31]. Further, among health care costs in the middle phase, the increase from none to the highest costs for hospitalization services, is especially noticeable. Other studies have not found similar pattern. This is likely explained by the fact that previous studies have collapsed a wider variety healthcare costs into one group (i.e. Jones et all, have one cost group for primary / home care, including nursing home care, while we report this separately) [20–22].

Furthermore, rehabilitation costs are found to be a substantial part of costs in early and middle HD and include specific HD rehabilitation programs offered to HD patients in these phases as part of secondary health care in Norway. Possibly they partly substitute needs for secondary care, specialists and hospital health care services.

Contrary to previously published studies, we investigated potential relationships between costs and socio-demographic and clinical characteristics. Associations found confirmed the strong effect of disease severity (disease phase). Being male was associated with lower total societal costs. This gender effect may be explained by men being more and longer in the work force in our sample.

### Strengths and limitations

To our knowledge this is the first study to include patients’ production loss complementing costs input related to informal care in order to estimate the societal costs related to HD. Including costs due to production loss in calculations of total societal costs emphasizes the importance of the economic burden for both the patient and caregiver and provide a more accurate outlook of HD for future policy decision making. In addition, the information collected in our study, could be used in an economic evaluation from a societal perspective, which would broaden the current healthcare perspective. The inclusion of productivity losses related to caregivers, as informal care is the most important cost driver in HD, could also have been an important factor to include in the estimation of social costs. Inclusion of productivity loss in future studies could provide more valid “real life” costs for HD and improve decision making. Furthermore, HRQoL values may also be reduced for caregivers, at least part of the disease trajectory, as balancing work life and providing informal care is challenging and a psychological burden. Future studies on the health and economic consequences of HD, should focus on a broader perspective on informal caregivers to account for the total HD burden.

One strength of this study lies on the use of individual patient data to calculate costs and HRQoL values avoiding the bias that comes from using external sources as basis for the analysis. The study population in the present study covers the whole spectrum of HD, with a relatively large number of patients in advanced stages compared to other studies, hence providing a more realistic estimation of HD costs.

Limitations of this study include the small sample size, limiting statistical power of associations between patient characteristics and costs or HRQoL. Furthermore, the cross-sectional design implies inability to make inferences of causal relationships between the investigated variables. Future studies on health economics should include larger samples and be based on longitudinal data. Moreover, information for pharmacological treatment, adaptations and aids, were not systematically recorded as part of this study contrary to other available studies on costs and economic burden [20–22]. Due to the lack of these data, total costs reported in this paper may still be considered an underestimation of the real societal costs due to HD.

## Conclusions

The present study reports data that may be used in modelling the cost effectiveness of new treatments for HD patients, informing stakeholders and policy makers. In line with previous studies, we found that disease severity (HD phase) is associated with decreased HRQoL and increased costs in middle and advanced phases of HD. Moreover, the present study highlights the important contribution of the informal care provided by partners, other family members or friends to the treatment of HD patients. These costs may be considered especially high considering that Norway is a welfare state. Although we included estimations of productivity loss, further efforts should be made to estimate productivity loss for informal caregivers, given the fact that they provide substantial care to their loved-ones. Given that the present study did not include estimations of costs for medication and aids, our results are likely still to be an underestimation of the total economic burden of HD in Norway. Based on general information about disease duration and care needed, it is important to consider that the health and economic burden for society as well as for the individual patient and his/her family members of HD, is likely to be present over many years.

## Supplementary Information

**Additional file 1.**

**Additional file 2.**

**Additional file 3.**

## Data Availability

The database created and analysed for this study can be made available from the corresponding author on reasonable request.

## Abbreviations

ADL: Activities of Daily Living
CSRI: Client Receipt Services Interview
DRG: Diagnosis Related Group
EQ-5D-3L: Three-level EuroQol five-dimentional questionnaire
FAS: Functional Assessment Scale
GP: General Practitioner
HD: Huntington’s Disease
HRQoL: Health-related quality of Life
IS: Independence Scale
KOSTRA: KOmmune STat RApportering (average costs in a Norwegian database with administrative information about municipal and county activities)
TFC: Total Functional Capacity
UHDRS: Unified Huntington’s Disease Rating Scale
