# Supplementary material for: Health related quality of life, service utilization and costs for patients with Huntington’s disease in Norway
Source: BMC Health Serv Res. 2022 Dec 14;22:1527. doi: 10.1186/s12913-022-08881-8 (PMC9753307; doi:10.1186/s12913-022-08881-8)
Supplement: Supplementary file 1 — Additional file 1. [file 12913_2022_8881_MOESM1_ESM.docx]

**Additional file 1.  *Workforce and earnings for patients with HD and the general population in Euro***

| Age | n | Full-Time (n) | Part-Time (n) | Workforce % | | Earnings | |
| --- | --- | --- | --- | --- | --- | --- | --- |
|  |  |  |  | HD | General | HD | General |
| < 30 | 1 | - | - | 0 | 84 | 0 | 22460,38 |
| 30-39 | 5 | 1 | 1* | 40 | 88 | 9911,50 | 26083,02 |
| 40-54 | 29 | 6 | 1** | 24 | 86 | 6719,34 | 29524,53 |
| 55-64 | 33 | 2 | 3*** | 15 | 69 | 2871,79 | 29615,09 |
| 65-74 | 14 | - | - | 0 | 13 | - | - |
| ≥75 | 4 | - | - | - | - | - | - |

Note. *= this patient had a 90% position. ** = this patient had a 60% position- ***= two patients had a 50% position and the other patient a 20% position.
