# Supplementary material for: Health related quality of life, service utilization and costs for patients with Huntington’s disease in Norway
Source: BMC Health Serv Res. 2022 Dec 14;22:1527. doi: 10.1186/s12913-022-08881-8 (PMC9753307; doi:10.1186/s12913-022-08881-8)
Supplement: Supplementary file 2 — Additional file 2. [file 12913_2022_8881_MOESM2_ESM.docx]

**Additional file 2**: *Six-month HD Costs in Euro’s divided across disease phase (belonging to Figure 1).*

| **Severity** | **Early** | | | | **Moderate** | | | | **Severe** | | | |
| --- | --- | --- | --- | --- | --- | --- | --- | --- | --- | --- | --- | --- |
| **Type of care** | **Mean** | **SD** | **Min** | **Max** | **Mean** | **SD** | **Min** | **Max** | **Mean** | **SD** | **Min** | **Max** |
| **Primary care** | 1274 | 2513 | 0 | 14683 | 1786 | 2064 | 66 | 6968 | 795 | 1730 | 0 | 9734 |
| **Home care** | 555 | 1619 | 0 | 9283 | 1746 | 2777 | 0 | 8491 | 3053 | 13729 | 0 | 76415 |
| **Nursing homes** | 0 | 0 | 0 | 0 | 0 | 0 | 0 | 0 | 26609 | 22987 | 0 | 4582 |
| **Specialists** | 160 | 351 | 0 | 1649 | 488 | 1380 | 0 | 5764 | 46 | 114 | 0 | 479 |
| **Rehabilitation** | 3405 | 3890 | 0 | 7718 | 5280 | 3686 | 0 | 7718 | 747 | 23119 | 0 | 7718 |
| **Secondary care** | 270 | 268 | 0 | 1054 | 472 | 772 | 0 | 2796 | 60 | 116 | 0 | 324 |
| **Hospital** | 0 | 0 | 0 | 0 | 4378 | 16645 | 0 | 72783 | 3572 | 10356 | 0 | 47325 |
| **Total Health care costs** | 5591 | 6227 | 0 | 24212 | 14150 | 19499 | 109 | 92491 | 35559 | 24950 | 331 | 93500 |
| **Informal care** | 2021 | 4210 | 0 | 17203 | 9605 | 13529 | 0 | 49837 | 76747 | 207772 | 0 | 1169811 |
| **Total care costs** | 4184 | 6714 | 161 | 26657 | 13768 | 14189 | 161 | 52104 | 110204 | 214497 | 375 | 1207547 |
| **Social worker** | 3 | 19 | 0 | 113 | 0 | 0 | 0 | 0 | 0 | 0 | 0 | 0 |
| **Out-of-pocket** | 69 | 48 | 0 | 242 | 159 | 450 | 0 | 2009 | 53 | 35 | 0 | 121 |
| **Production loss** | 13851 | 13806 | 0 | 29615 | 21436 | 13206 | 0 | 29615 | 22902 | 12573 | 0 | 29615 |
| **Total Societal costs** | 22005 | 17008 | 263 | 64439 | 40817 | 18960 | 246 | 81720 | 133222 | 216571 | 16978 | 1245283 |
