# Supplementary material for: Health related quality of life, service utilization and costs for patients with Huntington’s disease in Norway
Source: BMC Health Serv Res. 2022 Dec 14;22:1527. doi: 10.1186/s12913-022-08881-8 (PMC9753307; doi:10.1186/s12913-022-08881-8)
Supplement: Supplementary file 3 — Additional file 3. [file 12913_2022_8881_MOESM3_ESM.docx]

**Additional file 3**. Description (mean and standard deviation) of HD related costs (six-month period) expressed in Euro’s across disease phases presented for civil status.

| **Severity** | **Early** |  |  |  | **Moderate** | |  |  | **Severe** |  |  |  |
| --- | --- | --- | --- | --- | --- | --- | --- | --- | --- | --- | --- | --- |
| **Type of care** | **Married** | | **Single** | | **Married** | | **Single** | | **Married** | | **Single** | |
|  | Mean | SD | Mean | SD | Mean | SD. | Mean | SD | Mean | SD | Mean | SD |
| Primary care | 1476 | 3022 | 852 | 680 | 2373 | 2661 | 1134 | 843 | 1165 | 2514 | 472 | 265 |
| Home care | 274 | 491 | 1142 | 2748 | 1525 | 2800 | 1992 | 2899 | 6235 | 1957 | 70 | 247 |
| Nursing homes | 0 | 0 | 0 | 0 | 0 | 0 | 0 | 0 | 24441 | 23665 | 28642 | 22913 |
| Rehabilitation | 348 | 373 | 265 | 367 | 510 | 352 | 485 | 364 | 97 | 256 | 45 | 182 |
| Specialists | 71 | 215 | 347 | 499 | 627 | 1810 | 335 | 362 | 54 | 124 | 38 | 107 |
| Secondary care | 288 | 291 | 230 | 212 | 468 | 711 | 475 | 861 | 66 | 125 | 54 | 112 |
| Hospital | 0 | 0 | 0 | 0 | 7798 | 22892 | 578 | 1733 | 5928 | 14126 | 1365 | 4181 |
| **Total Health care costs** | 2457 | 3601 | 3012 | 4014 | 13307 | 16184 | 4991 | 3010 | 40132 | 27197 | 30685 | 22088 |
| Informal care | 2104 | 4600 | 1854 | 3499 | 8763 | 12217 | 10541 | 15557 | 127104 | 300225 | 32685 | 30096 |
| **Total care costs** | 4458 | 6923 | 4848 | 7618 | 22070 | 33261 | 15532 | 14462 | 176431 | 316646 | 63369 | 28338 |
| Social worker | 0 | 0 | 10 | 34 | 0 | 0 | 0 | 0 | 0 | 0 | 0 | 0 |
| Out-of-pocket | 661 | 54 | 74 | 32 | 247 | 620 | 61 | 34 | 52 | 41 | 53 | 30 |
| Production loss | 12578 | 13936 | 16512 | 13793 | 20368 | 14097 | 22621 | 12876 | 23674 | 12252 | 22177 | 13224 |
| **Total Societal costs** | 16531 | 13552 | 13589 | 17706 | 42438 | 32151 | 38154 | 19716 | 199198 | 318985 | 85547 | 32185 |
